# Lookback Exercise with Imported Crimean-Congo Hemorrhagic Fever, Senegal and France

**DOI:** 10.3201/eid1209.060002

**Published:** 2006-09

**Authors:** Arnaud Tarantola, Pierre Nabeth, Pierre Tattevin, Christian Michelet, Hervé Zeller, for the Incident Management Group

**Affiliations:** * Institut de Veille Sanitaire, Paris, France;; † Formerly of Institut Pasteur, Dakar, Senegal;; ‡ CHU Pontchaillou, Rennes, France;; § Centre National de Référence pour les Arboviroses, Lyon, France

**Keywords:** hemorrhagic fever virus, Crimean-Congo, occupational exposure, needlestick, hemorrhagic fevers, viral, travel, infection control, guidelines

## Abstract

A patient with suspected malaria was hospitalized successively in 2 hospitals, first in Dakar, Senegal, then in Rennes, France, where tests diagnosed Crimean-Congo hemorrhagic fever. An international incident management group was set up in France and Senegal, which traced 181 contacts and analyzed 50 samples from 3 countries. No secondary cases were identified clinically.

A case of imported Crimean-Congo hemorrhagic fever (CCHF) was diagnosed in France in November 2004. Clinical features are described elsewhere (*1*). The patient was a 60-year-old French woman who had been hospitalized on November 4, 2004, in Dakar, Senegal, for severe influenzalike illness that had lasted 3 days. As her condition worsened, she was repatriated to Rennes University Hospital, France, on November 14. Serum samples obtained November 15 were sent to the National Reference Center for Hemorrhagic Fevers (NRCHV, Institut Pasteur, Lyon, France). Tests for anti-CCHF–specific immunoglobulin (Ig) M antibody by enzyme-linked immunosorbent assay (ELISA) and reverse transcription–PCR were positive. Tests for anti-CCHF IgG were negative.

After the NRCHV telephoned the diagnosis on November 22 (18 days after the date of first admission and 8 days after admission at Rennes University Hospital), control measures were taken immediately by an ad hoc group of persons and institutions responsible for prevention and control of infectious diseases. Contract tracing at Rennes Hospital was coordinated by the hospital's infection control committee, which identified all contacts among healthcare workers and persons who had handled or analyzed the patient's samples. The regional health authorities and epidemiologists from the Cellule Interrégionale d'Epidémiologie (CIRE) identified and followed-up all contacts in the patient's family. The Institut de Veille Sanitaire (InVS) documented the sequence of medical management and opportunities for accidental blood exposure to blood (*2*), informed all the healthcare worker teams that managed the patient's care, and coordinated efforts with the Institut Pasteur of Dakar in Senegal. The NRCHV made and reported the diagnosis. In Senegal, the Institut Pasteur was in charge of the investigation and contact tracing, which were carried out by epidemiologists with the assistance of entomologists and virologists.

The maximum duration of viremia was estimated to be 12 days, and the disease incubation period was estimated at 10 days (*3**,**4*). The group followed up potentially exposed persons for 10 days after a possible contact with the patient from November 4 through November 16, or 10 days after handling blood or tissue samples taken through November 16. A contact person was defined as anyone who was in direct contact (for example, family visitor, healthcare worker) with the patient or with samples taken from the patient between these dates. Contact persons did not undergo serologic screening, but all were informed of the need to self-monitor and were each followed up daily by a clinician either directly or by phone.

No accidental blood exposure was documented during healthcare procedures or handling of patient samples by any of the healthcare teams. Table 1 presents a summary of the sequence of events and occasions for secondary transmission. In Senegal, the patient had been admitted to a double room where she remained throughout her stay at hospital 1, except on the evening of her transfer to hospital 2. She had undergone several blood tests, received infusions, and had several visits from friends and colleagues. She had been transferred once to a dental clinic for bleeding gums. In hospital 2, she had been placed in a single room in the intensive care unit (ICU). Isolation procedures had been observed, and she had received no visitors. A central intravenous line had been placed, and blood samples had been obtained on several occasions. No isolation precautions were observed in the ambulance that had transferred her to the ambulance plane to Rennes. On the plane the patient's condition had been managed by a German medical team who had transfused whole blood through an existing catheter. The team had worn gloves but no masks. On arrival, she was transferred by ambulance from the Rennes airport to Rennes University Hospital.

**Table 1:** Synopsis of phases, risks, and management of case of Crimean-Congo hemorrhagic fever*

Institution/phase	Place/date	Contacts, persons at risk	Measures	No. contacts (N = 181)	Responsible
Incubation phase	Senegal/Oct–Nov 2	Contacts in Saly, Senegal	SOS Médecins Saly informed.	2	IPD and DIT-InVS
Before hospitalization	Dakar, Senegal/Nov 2–4	Radiologist and his family	Radiologist informed	5	IPD and DIT-InVS
Hospitalization 1	Dakar, Senegal/Nov 4–10	HCWs and laboratory personnel	Head physicians informed and agreed to verify staff health status	20	IPD and DIT-InVS
		Patients who shared the room	Head physicians informed and agreed to verify patient health status	4	IPD and DIT-InVS
			Other physicians in health clinics welcoming expatriates informed		IPD and DIT-InVS
		Dental appointment	Dentist informed	1	IPD and DIT-InVS
		Visitors (friends)	Physician and radiologist informed	10	IPD and DIT-InVS
Ambulance transport 1	Dakar, Senegal/Nov10	Personnel who transported the patient	Head physicians informed and agreed to verify staff health status	2	IPD and DIT-InVS
Hospitalization 2	Dakar, Senegal/Nov 10–13	HCWs who provided care	Head physicians informed and agreed to verify staff health status	25	IPD and DIT-InVS
Ambulance transport 2	Dakar, Senegal/Nov 13	Personnel who transported the patient	Head physicians informed and agreed to verify staff health status	2	IPD and DIT-InVS
Medical transport plane	Plane Dakar-Paris/Nov 13–14	German team who provided care	Head physician informed and agreed to verify staff health status; German health authorities informed	2	DIT-InVS
Ambulance transport 3	Rennes, France/Nov 14	Personnel who transported the patient	Identification of contacts. Daily clinical follow up of contacts until Nov 26	2	Ddass 35 and CIRE Quest
Hospitalization 3	Rennes, France/Nov 14–24	ICU personnel who provided care	Identification of contacts. Daily clinical follow up of contacts until Nov 26	34	C-Clin Ouest, Ddass 35 and CIRE Ouest
		ID personnel who provided care	Identification of contacts. Daily clinical follow up of contacts until Nov 26	10	C-Clin Ouest, Ddass 35 and CIRE Ouest
		Personnel who transported the patient between wards	Identification of contacts. Daily clinical follow up of contacts until Nov 26	3	C-Clin Ouest, Ddass 35 and CIRE Ouest
		Laboratory personnel	Identification of contacts. Daily clinical follow up of contacts until Nov 26; identification/tracing of samples	50	C-Clin Ouest, Ddass 35 and CIRE Ouest
		Patient's family who visited	Identification of contacts. Daily follow up of contacts by telephone until Nov 26	9	C-Clin Ouest, Ddass 35 and CIRE Ouest

*HCW, healthcare workers; ICU, intensive care unit; ID, infectious diseases department; IPD, Institut Pasteur, Dakar; DIT, Département International et Tropical; InVS, Institut de Veille Sanitaire; CIRE, Cellule Interrégionale d'Epidémiologie.

While in ICU in the Rennes Hospital, isolation precautions were observed (strict adherence to standard precautions [5]; masks; single room). Visitors, however, were allowed. The masks were simple surgical masks, and the room was not a negative-pressure room. Blood samples were obtained repeatedly.

As her clinical status improved, the patient was transferred to a single room in the infectious disease department 2 days later. Again, standard precautions were strictly observed. The first biologic samples were sent to the laboratory in observance with standard procedures but with no particular additional precautions.

The group identified 71 patient contacts in Senegal and 2 (air ambulance transfer personnel) in Germany (Table 1). At the Rennes Hospital, 44 staff members in ICU or infectious disease departments, 50 laboratory personnel, and 3 persons responsible for bedside radiography or patient transfers were identified and contacted (Table 2). A total of 50 samples were identified and later destroyed.

**Table 2:** Possible patient contacts among Rennes Hospital personnel, by occupation

Work unit	Nurses	Physicians/medical students	Nurses' aides	Ambulance staff	Technicians	Total
Intensive care unit	7	10	17	0	0	34
Infectious diseases department	3	4	3	0	0	10
Laboratories	0	1	10	0	39	50
Transportation and radiology	0	0	0	2	1	3
Total	10	15	30	2	40	97

Within 36 hours, the international team had identified 181 contacts among healthcare workers, visitors, fellow patients, and ambulance drivers in France, Germany, or Senegal and immediately informed the head physicians of each team that had cared for the patient since the date of symptom onset. No accidental blood exposure was identified, but extensive casual contact was documented. No secondary cases were identified clinically in any of the 3 countries.

The first symptomatic CCHF case in Senegal was identified in 2003 (*6*), but seroprevalence studies indicated that CCHF virus has been circulating there since 1969 (*7**,**8*). Another unrelated patient with a confirmed case, who had stayed in the same coastal area as our index case-patient in late November 2004, also became ill and exhibited fever and severe bleeding. She died 4 days later, causing no secondary cases among >4 healthcare workers and 4 friends or colleagues who cared for her, including one who sustained extensive accidental blood exposure but received ribavirin (P. Nabeth, pers. comm.).

This investigation of a case of imported viral hemorrhagic fever in France raised several issues. First, the patient did not have Lassa fever, unlike the diagnosis in most imported cases of viral hemorrhagic fever in industrialized countries (*9*). Second, the illness did not occur in a major French metropolitan center with an international airport that was home to a large community of migrant workers such as Paris, Lyon, or Marseille. The third issue is that of transmission and prevention of secondary cases. Suspected diagnosis was not announced by the team in Senegal before transfer. Therefore only standard precautions were observed during transfer or during this patient's care or the management of her blood and tissue samples. The patient was transferred and hospitalized in France 10 days after the onset of the first clinical signs. The absence of secondary cases could therefore be due to low levels of viremia because by the time the patient arrived in France the infection was nearing its end. However, no cases were described in Senegal either, where the patient was also hospitalized and also underwent invasive procedures in less stringent isolation conditions during a phase of high levels of viremia. The observance of standard precautions was therefore sufficient to prevent clinical secondary cases in Senegal, Germany, and France, as extensively described in developing or industrialized country settings (*10**,**11*). Observance of standard precautions has greatly improved in France and elsewhere during the last decade, as shown by the documented reduction of occupational infections and accidental blood exposures in French hospitals (*12**,**13*). These factors may have played a decisive role in the absence of clinically patent secondary transmission.

Hemorrhagic fever viruses leave no room for complacency. The risk for transmission and available means of prevention, however, are becoming increasingly well documented. This first imported case of CCHF in an industrialized country should lead French health authorities to review available evidence and reexamine the justification and applicability of the existing, stringent recommendations for the management of many patients in whom viral hemorrhagic fever is one of several possible diagnoses.

## References

[1] Jaureguiberry S, Tattevin P, Tarantola A, Legay F, Tall A, Nabeth P, Imported Crimean-Congo hemorrhagic fever. J Clin Microbiol. 2005;43:4905–7. 10.1128/JCM.43.9.4905-4907.2005 16145173 PMC1234090

[2] Centers for Disease Control and Prevention. Guidelines for the prevention of transmission of human immunodeficiency virus and hepatitis B virus to health-care and public safety workers. MMWR Morb Mortal Wkly Rep. 1989;38:S63–87.

[3] Swanepoel R. Nairovirus infections. In: Porterfield JS, editor. Exotic viral infections. London: Chapman and Hall; 1995. p. 285–93.

[4] Data from studying etiology, laboratory diagnosis and immunology of Crimean hemorrhagic fever: questions of ecology of the viral agent. Inst Polio Virusn Entsefalitov Akad Med Nauk SSSR. [article in Russian]. (English version, US Naval Medical Research Unit No-3–T1152). 1971.

[5] Garner JS. Guideline for isolation precautions in hospitals. The Hospital Infection Control Practices Advisory Committee. Infect Control Hosp Epidemiol. 1996;17:53–80. 10.1086/647190 8789689

[6] Nabeth P, Thior M, Faye O, Simon F. Human Crimean-Congo hemorrhagic fever, Senegal. Emerg Infect Dis. 2004;10:1881–2. 15565746 10.3201/eid1010.040586 PMC3323271

[7] Chunikhin SP, Karaseva PS, Tofflib R, Roben I, Kornet M. Results of a serologic study of the cycles of circulation of several arboviruses in the Republic of Senegal (West Africa) [article in Russian]. Vopr Virusol. 1971;16:52–5. 5105875

[8] Wilson ML, LeGuenno B, Guillaud M, Desoutter D, Gonzalez JP, Camicas JL. Distribution of Crimean-Congo hemorrhagic fever viral antibody in Senegal: environmental and vectorial correlates. Am J Trop Med Hyg. 1990;43:557–66. 2122750 10.4269/ajtmh.1990.43.557

[9] Macher AM, Wolfe MS. Historical Lassa fever reports and 30-year clinical update. Emerg Infect Dis. 2006;12:835–7. 16704848 10.3201/eid1205.050052 PMC3374442

[10] Athar MN, Khalid MA, Ahmad AM, Bashir N, Baqai HZ, Ahmad M, Crimean-Congo hemorrhagic fever outbreak in Rawalpindi, Pakistan, February 2002: contact tracing and risk assessment. Am J Trop Med Hyg. 2005;72:471–3. 15827289

[11] Peters CJ, Jahrling PB, Khan AS. Patients infected with high-hazard viruses: scientific basis for infection control. Arch Virol Suppl. 1996;11:141–68. 8800796 10.1007/978-3-7091-7482-1_13

[12] Abiteboul D, Lamontagne F, Lolom I, Tarantola A, Descamps JM, Bouvet E, Incidence des accidents exposant au sang chez le personnel infirmier en France métropolitaine, 1999–2000: résultats d'une étude multicentrique dans 32 hôpitaux. Bull Epidemiol Hebd. 2002;51:259.

[13] Tarantola A, Golliot F, Astagneau P, Fleury L, Brücker G, Bouvet E, Occupational blood and body fluids exposures in health care workers: Four-year surveillance from the Northern France Network. Am J Infect Control. 2003;31:357–63. 10.1016/S0196-6553(03)00040-3 14608303

